# The Impact of Periconceptional Alcohol Use on the Etiology of Eating Disorders

**DOI:** 10.1192/j.eurpsy.2025.1386

**Published:** 2025-08-26

**Authors:** M. S. Artemieva, R. A. Suleymanov, I. E. Danilin

**Affiliations:** 1 Psychiatry and Medical Psychology, RUDN University, Moscow, Russian Federation

## Abstract

**Introduction:**

The adverse social impact of parental alcoholism on children’s development is well known and is primarily associated with difficulties in social adaptation, a higher risk of anxiety, depression, personality disorders, etc. At the same time, in the genesis of neuropsychiatric disorders in such children, parental alcoholism can be both a socio-psychological and biological factor, through its impact on germ cells, the developing fetus, and postnatal development. The dates of birth of the studied cohort of patients fell in the period of the late 1970s - first half of the 1980s - years of steady growth in alcohol consumption in the late Soviet Union, when, according to the USSR State Statistics Committee, the average resident of the USSR consumed (excluding production by the population, primarily moonshine) an average of 10.7 liters of pure alcohol per capita. Soviet tradition prescribed the mandatory presence of alcoholic beverages on the holiday table. At the same time, the longest and most widespread alcohol consumption in the USSR occurred on summer vacations and official state holidays, for which extraordinary non-working days were established. Along with traditional summer holidays (July and August), such periods were, the second half of February – the first half of March (passing from one to another, the celebration of February 23 (Soviet Army Day and March 8), the end of December/the non-working first half of January (a ten-day celebration of the New Year), the celebration of Revolution Day (the first half of October).

**Objectives:**

To investigate the possible role of increased alcohol abuse during the periods of conception of children who will later suffer from nervous anorexia and bulimia.

**Methods:**

The approximate dates of conception (considering the terms of full-term pregnancy and the date of birth) of patients (N=191) with eating disorders (AN and NB) born before 1991 were analyzed in relation to the periods of traditional mass alcohol consumption in the late USSR.

**Results:**

The frequency of conception of future patients with eating disorders in the study group during periods of traditionally stable growth in alcohol consumption was 1.4–1.7 times higher (p < 0.01) than in other periods. A clear pattern emerged from the analyses. That is, eating disorders, like other chronic psychiatric diseases, are the product of multiple factors, however, alcohol abuse during conception clearly increases the risk of having daughters with AN and BN.

**Conclusions:**

In conclusion, periconceptional alcohol consumption appears to significantly elevate the risk of offspring developing eating disorders, specifically anorexia nervosa and bulimia nervosa. The study reveals a marked increase in conception during peak alcohol consumption periods, suggesting alcohol’s potential role in the etiology of these disorders.

**Disclosure of Interest:**

None Declared

